# Loganetin and 5‐fluorouracil synergistically inhibit the carcinogenesis of gastric cancer cells via down‐regulation of the Wnt/β‐catenin pathway

**DOI:** 10.1111/jcmm.15932

**Published:** 2020-10-24

**Authors:** Huaixiang Zhou, Xiaoge Hu, Na Li, Guangyan Li, Xiaotian Sun, Feimin Ge, Jiahong Jiang, Jingchun Yao, Dongsheng Huang, Liu Yang

**Affiliations:** ^1^ Department of Medical Oncology Zhejiang Provincial People’s Hospital People’s Hospital of Hangzhou Medical College Hangzhou China; ^2^ Key Laboratory of Tumor Molecular Diagnosis and Individualized Medicine of Zhejiang Province Zhejiang Provincial People’s Hospital People’s Hospital of Hangzhou Medical College Hangzhou China; ^3^ State Key Laboratory of Genetics Manufacture Technology of Chinese Traditional Medicine Lunan Pharmaceutical Group Co., Ltd Linyi China; ^4^ Department of Internal Medicine Clinic of August First Film Studio Beijing China

**Keywords:** EMT, gastric carcinoma, loganetin, stem‐like properties, β‐catenin

## Abstract

Although most gastrointestinal tumours are sensitive to 5‐fluorouracil (5FU), drug resistance is commonly occurred after 5FU therapy in gastric cancer (GC). Loganetin is the primary active compound in *Cornus officinali*. However, the synergetic effects of loganetin and 5FU on GC remain unknown. Here, we investigated the synergetic effects and the underlying mechanism of loganetin and 5FU on proliferation, stem‐like properties, migration, and invasion of GC both in vitro and in vivo. We found that loganetin alone inhibited the proliferation, stem‐like properties, migration and invasion of GC cells in vitro. Importantly, the loganetin remarkably enhanced the anti‐cancer effect of 5FU on GC cells and the Wnt/β‐catenin pathway might be involved in this process. Animal experiments further confirmed the synergistic effects of 5FU and loganetin on inhibiting cell growth and metastasis of GC. These results suggested that loganetin could synergistically increase the effect of 5FU against GC, which sheds light on effective combinational drug strategies for GC treatment.

## 1 INTRODUCTION

Gastric cancer (GC) is a malignant cancer, remaining the fifth most frequently encountered tumour type and the third leading cause of death.^1^ Globally, 14 million people are diagnosed with cancer each year, and more than a quarter of them are gastrointestinal tumours.^2^ Although the global mortality rate of GC has been declining in recent years, the number of new cases is still high in developing countries like China.^3^, ^4^ The first‐line chemotherapy regimen recommended for advanced GC patients contains the platinum‐based fluorouracil, and the second‐line treatments including docetaxel, paclitaxel or irinotecan.^5^ However, while chemotherapy drugs kill cancer cells, they also lead to the emergence of drug resistance.^6^ Most gastrointestinal tumours are sensitive to 5‐fluorouracil (5FU) treatment.^7^ However, GC patients generally have drug resistance after 5FU treatment.^8^, ^9^ Therefore, it is necessary to study the combination of anti‐cancer drugs that effectively synergize with 5FU, improving the anti‐cancer therapeutic effect.

The synergy of therapeutic drugs is an active and exciting area of research in the field of tumour chemotherapy, as it represents a strategy with the potential to effectively overcome drug resistance.^10^ The combination of anti‐tumour drugs with different mechanisms of action can usually enhance the efficacy of these drugs, reduce the necessary dosage, provide a more effective and advanced solution for the clinical treatment of tumours, and improve the pain reduction of patients.^11^ *Cornus officinalis* is a rare medicinal plant ^12^with immunomodulatory and anti‐tumour effects which has been confirmed by modern pharmacological research.^13^, ^14^ The primary active ingredient in *C. officinalis* is loganin,^15^ which has been reported to exert its pharmacological effects via being metabolized by the gastrointestinal flora into loganetin.^16^ We hypothesized that loganetin may potentially facilitate the anti‐tumour effects of *C. officinalis* by altering key signalling pathways in tumour cells.

In the present study, we evaluated the effect of loganetin alone or together with 5FU on GC proliferation, stem‐like properties and EMT activity and explored the possible molecular mechanism of the anti‐cancer effect of loganetin combined with 5FU. These findings highlight the therapeutic potential of chemotherapeutic drugs combination in GC treatment.

## 2 MATERIALS AND METHODS

### 2.1 Cell lines and culture

The HGC27 and MGC803 human GC cell lines were purchased from the Cell Bank of the Chinese Academy of Sciences. All cell lines in this study were authenticated using STR DNA fingerprinting by Shanghai Biowing Applied Biotechnology Co.,Ltd.; Mycoplasma infection was detected using a Mycoplasma Stain Assay Kit (Beyotime). HGC27 cells and MGC803 cells were grown using RPMI 1640 (Hyclone) containing 10% FBS (BI), 100 U/mL penicillin and 100 μg/mL streptomycin (Gibco) in an incubator at 37°C and 5% CO_2_. For sphere formation assays, cells were grown and maintained in DME/F‐12 medium supplemented with N‐2 Supplement, 10 ng/mL bFGF and 20 ng/mL EGF (Gibco), along with penicillin/streptomycin.

### 2.2 Cell survival assay

A CCK8 assay was used to assess cell survival. HGC27 or MGC803 cells were seeded into 96‐well plates overnight and subsequently treated with the compounds of interest for an additional 24 hours. Next, the CCK8 solution (Bimake.Com) was added to the wells, and then incubated in the dark. At the indicated time points, a Multiskan Spectrum (Thermo Electron Corporation) device was used to assess the 450 nm absorbance values. All experiments were repeated in triplicate.

### 2.3 Colony formation assay

Cells were plated in six‐well plates, and then treated with 5FU, loganetin or the combination solution for 24 hours, after which ~1000 cells from each treatment condition were plated per 60‐mm dish. Media and drugs were changed every 3 days. After 15 days, colonies were stained and imaged. Stained cells were then extracted with 10% acetic acid, and measure the absorbance at 450 nm as described above.

### 2.4 Flow cytometry

To measure apoptosis, HGC27 or MGC803 cells were treated with 5FU, loganetin or both for 24 hours, followed by analysing with an Annexin V‐FITC/PI Apoptosis Detection Kit (Multi Sciences) based on the provided instructions. For surface staining, 1 × 10^6^ cells were stained with FITC‐conjugated anti‐CD44 (BioLegend) at 4°C for 30 minutes. Samples were then washed. All the data were obtained and analysed using Novo Express software (ACEA Biosciences, Inc).

### 2.5 qRT‐PCR

Trizol (Life) was used to extract HGC27 cell or MGC803 RNA based on the provided protocols. The PrimeScript RT reagent Kit with gDNA Eraser (Takara) was used to generate cDNA based on the provided directions. qRT‐PCR was then used to measure mRNA expression with a TB Green Premix Ex Taq II (Tli RNaseH Plus, Takara) per the provided directions. GAPDH and gene‐specific primers were used for these analyses (Table S1). The Bio‐Rad CFX Manager Software (Bio‐Rad) was used to analyse data.

### 2.6 Western blotting

Prepare the total lysate or nuclear lysate as in the previous study according to the designated processing method. Similarly, lysates extracted from tumour samples of treated mice were also collected and analysed as described in previous reports. Similarly, lysates from treated mouse tumour samples were collected and analysed as in previous reports.^17^ Nuclear and cytoplasmic proteins from HGC27 cells or MGC803 cells were extracted with Minute™ cytoplasmic and nuclear extraction kits (Invent Biotechnologies, Inc) based on the provided protocols.^18^

### 2.7 Transwell chamber assays

For invasion assays, 1 × 10^5^ cells in serum‐free medium were plated in the upper chamber of a 24‐well Transwell plate (Corning) that was coated with Matrigel (Corning). In the lower chamber, RPMI 1640 containing 10% FBS was added. After a 24 hours incubation period, HGC27 and MGC803 cell migration were assessed, and 0.1% crystal violet was used to stain cells, with five random visual fields counted via microscopy.

### 2.8 Immunofluorescent staining

β‐catenin expression was assessed via immunofluorescent staining in HGC27 and MGC803 cells. First, cells were fixed in 4% paraformaldehyde for 20 minutes. Then, after a 10 minutes permeabilization within 0.2% Triton X‐100 and blocking in 10% Normal Donkey Serum (NDS) for 60 minutes at room temperature, cells were incubated with anti‐β‐catenin rabbit polyclonal antibodies (CST) overnight at 4°C. Then the cells were washed thrice in PBS for 5 minutes each and incubated with Cy3‐conjugated donkey anti‐rabbit secondary antibodies (Sangon Biotech) for 1 hour at room temperature. Then the cells were washed thrice in PBS for 5 minutes each. Subsequently, cells were mounted using DAPI‐Fluoromount‐G (Southern Biotech) and photographed using a Confocal Microscope (Leica Microsystems).^19^

### 2.9 Luciferase vector transduction

HGC27 cells were stably transduced with luciferase‐encoding lentiviral overexpression vectors according to the manufacturer’s protocol (Shanghai Genechem Co., Ltd). Seed 5 × 10^4^ cells in one well of six‐well plate, and then add virus mixture (500 μL complete DMEM containing virus + 500 μL complete DMEM containing polybrene) in the following day. At 3 days, change medium to complete medium and culture for 1 more day. Finally, 2 μg/mL puromycin (Sigma‐Aldrich) was added to the cells to screen for positive cells.

### 2.10 In vivo experiments

All animal experiments were approved by the Institutional Animal Care and Use Committee (IACUC), Zhejiang Provincial People's Hospital. All procedures were in compliance with the Guidelines for the Care and Use of Laboratory Animals (National Academies Press, Washington, DC). All experimental animals were kept under a 12 hours light/ dark cycle in an SPF animal facility at Zhejiang Academy of Medical Sciences. The Institutional Review Board of Zhejiang Academy of Medical Sciences approved all housing and surgical procedures. In our experiment, the animals used were 4‐6 weeks old female BALB/c nude mice, from the Model Animal Research Center of Nanjing University.

To evaluate the value of 5FU, loganetin, or its combination in the treatment of GC xenografts in vivo, MGC803 cells were subcutaneously injected (1 × 10^6^ cells/mouse) into these animals. Once xenografts were 2 mm in diameter, 5FU (100 mg/kg) was injected i.p. once weekly from day 6 to 30; in addition, loganetin (20 mg/kg) was injected i.p. five times per week from day 6 to 30. In the combination group, the two drugs were administered separately, with a time interval of 48 hours. The control group was given normal saline. Tumour volumes were determined as follows: volume (mm^3^) = length × width^2^ × 0.5. On day 30, the xenografts were excised, weighed and imaged.

The effects of 5FU, loganetin and the combination treatment on GC metastasis were evaluated as follows. A suspension of HGC27‐luc cells was prepared at 1 × 10^7^ cells/mL. 200 μL of the cells were injected into the tail vein of nude mice, after which 5FU (100 mg/kg) was injected intraperitoneally once a week; loganetin (20 mg/kg) was injected intraperitoneally five times per week; or, for the combination group, the two drugs were administered separately, with a time interval of 48 hours. The control group was intraperitoneally injected with the same amount of normal saline. Animal body weight was recorded every day. Two weeks later, an in vivo fluorescence imaging system was used to assess tumour growth and metastasis. On the fourth week, the livers were excised, embedded in paraffin and assessed via haematoxylin‐eosin (H&E) staining.

### 2.11 Statistical analyses

GraphPad Prism 5.0 (GraphPad Software) was used for all analyses. Data were shown as means ± SD. Multiple groups were compared with the LSD test by one‐way analysis of variance, while two groups were compared by unpaired Student’s *t* test. *P* < .05 was the threshold of significance.

## 3 RESULTS

### 3.1 Synergistic inhibitory effects of 5FU and loganetin on human gastric cancer cell lines

**FIGURE 1:**
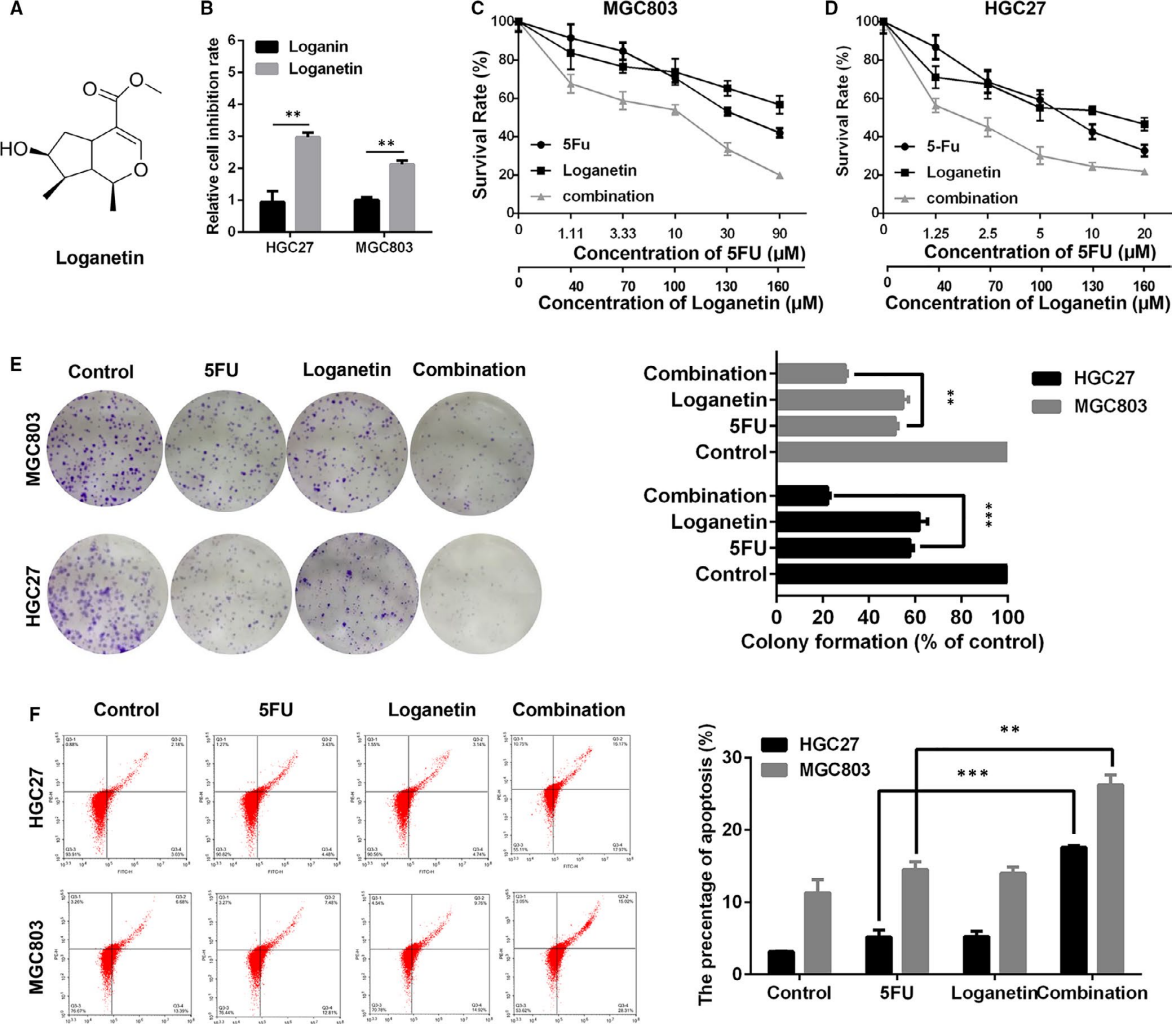
5FU and loganetin combine to cause cytotoxicity in HGC27 and MGC803 cells. (A) The molecular formula of loganetin. (B) The relative inhibition rate of HGC27 and MGC803 cells treated with loganin and loganetin (70μM) for 48 hours. (C and D) HGC27 and MGC803 cells were treated using the indicated doses of 5FU, loganetin, or a combination of the two for 48 hours, and survival was assessed via CCK8 assay. (E) HGC27 and MGC803 cells were exposed to 5FU (1.11 μM/ 1.25 μM), loganetin (40 μM), or both for 15 days, after which colony formation was assessed via crystal violet staining. A representative colony is on the left, while colony formation rates are on the right. (F) HGC27 and MGC803 cells were treated with 5FU (3.33 μM/ 2.5 μM), loganetin (70 μM), or both for 48 hours, and Annexin V/PI staining was used to assess apoptosis via flow cytometry. All statistical results were repeated for three times. * *P*<0.05; ** *P*<0.01; *** *P*<0.001

Loganetin (Figure 1A) is a natural product derived from *C. officinalis*.^20^ We found that at the same concentration, the inhibition of cell viability of loganetin (Figure 1B) on GC cells was about three times higher than that of loganin. Therefore, we focused on loganetin, investigating its potential synergy with 5FU on proliferation of human GC cell lines including HGC27 and MGC803. We found that the combination of 5FU and loganetin markedly reduced cell viability compared with either agent alone (Figure 1C,D). We further established combination index (CI) values at a range of 5FU and loganetin doses in order to gain more insight into whether these agents exhibited a synergistic effect on cancer cells by CalcuSyn. CI values <0.90 are consistent with synergy. In the present study, we found the CI values were <0.90 in both GC cell lines, indicating a synergistic function of 5FU and loganetin (Table S2). The appropriate concentrations for subsequent assays were selected based on GC cell activity and CI values.

We next assessed the anti‐cancer efficacy of this drugs combination by colony formation assay, revealing that HGC27 and MGC803 cells treated with both 5FU and loganetin survived to a lesser extent than those cells treated with 5FU or loganetin alone, even at relatively low concentrations (1.11 μmol/L 5FU and 40 μmol/L loganetin for HGC27 cells, 1.25 μmol/L 5FU and 40 μmol/L loganetin for MGC803 cells; Figure 1E, left). Colonies were then extracted using 10% acetic acid to assess their clonogenicity, revealing a more substantial reduction in colony formation upon combination treatment relative to monotherapeutic treatment (Figure 1E, right). We next assessed the apoptosis of HGC27 and MGC803 cells after 24 hours of exposure to 5FU, loganetin, or both (3.33 μmol/L 5FU and 70 μmol/L loganetin for HGC27 cells, 2.5 μmol/L 5FU and 70 μmol/L loganetin for MGC803 cells) in order to assess apoptotic cell death based on flow cytometry after Annexin V/PI staining (Figure 1F). Apoptosis rates of four groups including control, 5FU, loganetin and combination in HGC27 cells were 3.03%, 4.48%, 4.74% and 17.97%, respectively, while those rates in MGC803 cells were 13.39%, 12.81%, 14.92% and 28.31%, respectively. Together, these findings suggested the combination of 5FU and loganetin synergistically promoting GC cell apoptosis more effectively than either agent alone.

### 3.2 Synergetic effects of loganetin and 5FU on the stem‐like properties of HGC27 and MGC803 cells

**FIGURE 2:**
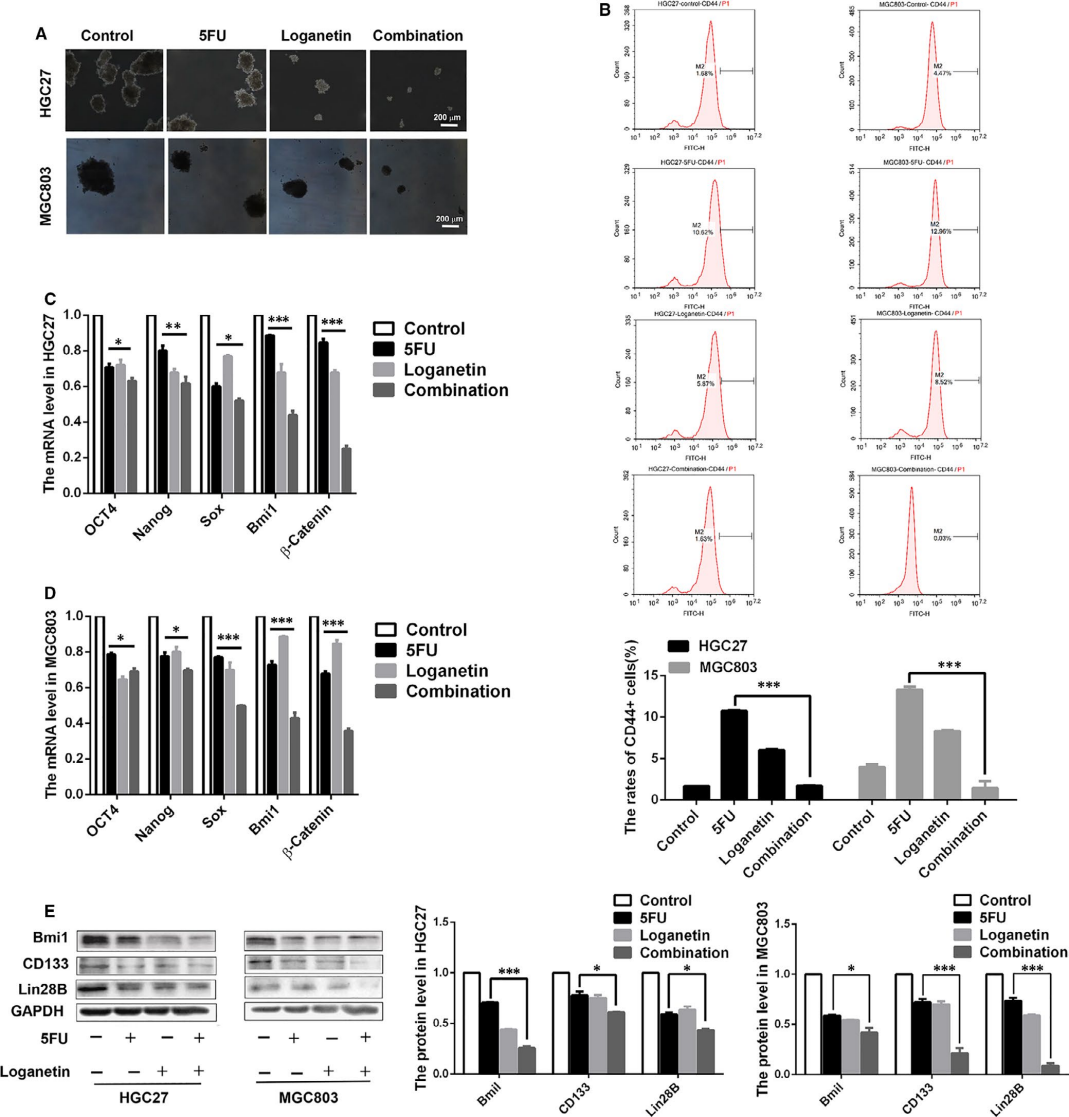
Loganetin and 5FU together affect the stemness of HGC27 and MGC803 cells. (A) A tumorsphere formation assay revealed that the combination of 5FU (3.33 μM/ 2.5 μM) and loganetin (70 μM) reduced tumorsphere number and size for both HGC27 and MGC803 cells relative to 5FU treatment alone on day 14. (B) HGC27 and MGC803 cells were exposed to 5FU (3.33 μM/ 2.5 μM), loganetin (70 μM), or both for 24h. After which, flow cytometry revealed fewer CD44+ cells in the combination‐treated group relative to the 5FU treatment group. qPCR (C and D) and Western blotting (E) showed that the combination of 5FU (3.33 μM/ 2.5 μM) and loganetin (70 μM) inhibited stemness‐related genes in HGC27 and MGC803 cells with stem‐like properties. All statistical results were repeated for three times. * *P*<0.05; ** *P*<0.01; *** *P*<0.001

Using spheroid formation experiments, we found that there was a decrease in HGC27 and MGC803 spheroid formation (Figure 2A) after treatment with the combination of 5FU and loganetin. CD44 is the marker of GC stem cells.^21^ When we measured the frequency of CD44^+^ GC cells after drug treatments, we found that rates of CD44^+^ GC cells in the control, 5FU, loganetin and combination groups for HGC27 cells were 1.68%, 10.62%, 5.87% and 1.63%, respectively, while rates in MGC803 cells were 4.47%, 12.96%, 8.52% and 0.03%, respectively (Figure 2B). This indicates that the 5FU treatment of GC cells can enrich the stem‐like cells, while the combination of 5FU and loganetin can inhibit this phenotype. Using RT‐qPCR to detect the expression of stemness‐related gene expression, we found that the combination of 5FU and loganetin significantly down‐regulated the stemness‐related genes *OCT4*, *Nanog*, *Sox*, *Bmi1* and *β‐catenin* (Figure 2C,D). The results of Western blotting showed that the combination of 5FU and loganetin significantly down‐regulated Bmi1, CD133 and Lin28B proteins (Figure. 2E). Together, these findings indicate that the combination of 5FU and loganetin can inhibit the stem‐like properties of HGC27 and MGC803 cells in GC.

### 3.3 Synergetic effects of loganetin and 5FU on the invasion and migration of HGC27 and MGC803 cells

**FIGURE 3:**
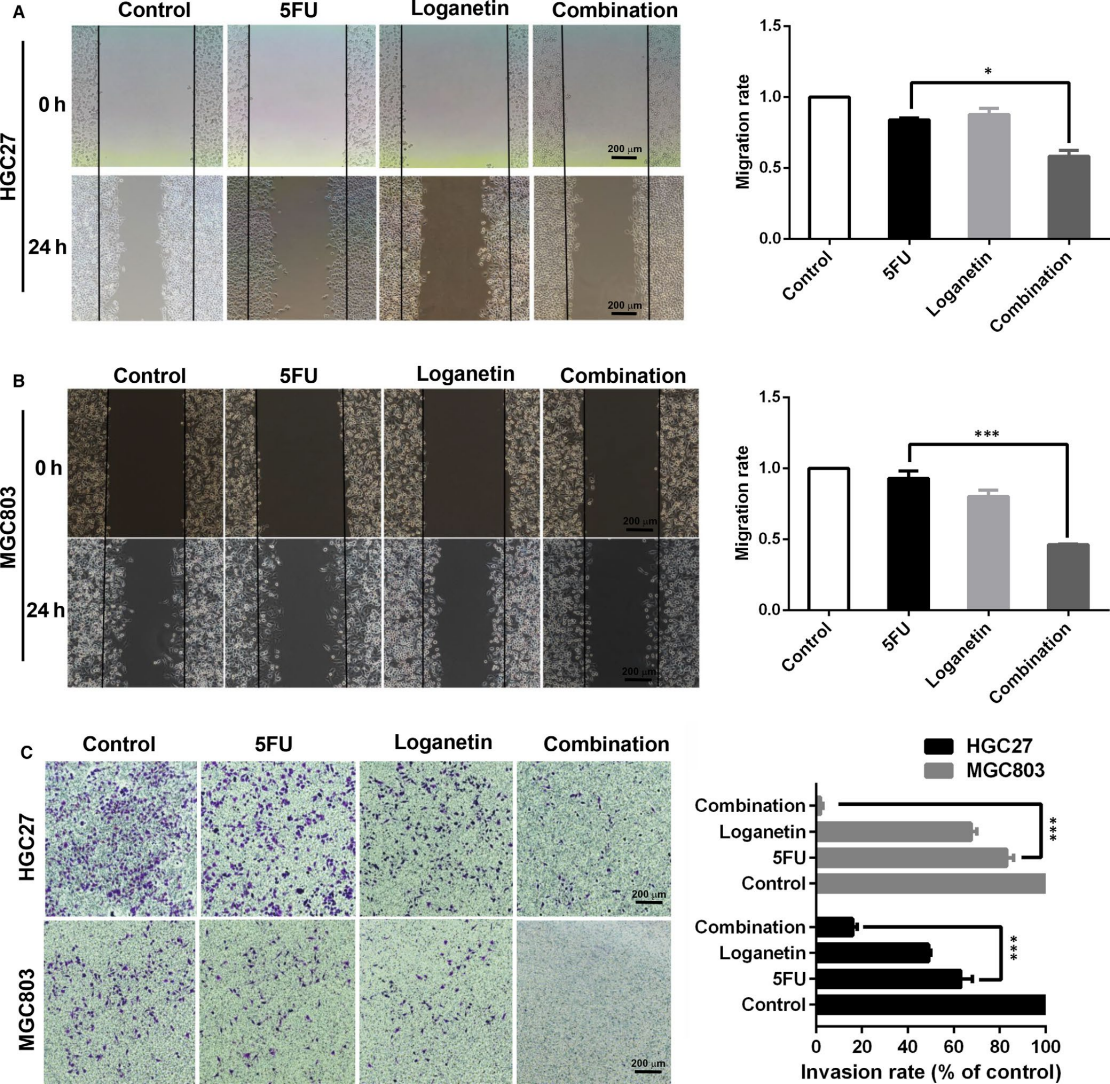
Combination of 5FU and loganetin inhibited cell migration and invasion of HGC27 and MGC803 cells. (A and B) HGC27 and MGC803 cells were exposed to 5FU (3.33 μM/ 2.5 μM), loganetin (70 μM), or both for 24h. A scratch test revealed that the combination of 5FU and loganetin inhibited HGC27 and MGC803 cell migration relative to 5FU alone. (C) Transwell chamber assays revealed that the combination of 5FU and loganetin inhibited cell invasiveness for both HGC27 and MGC803 cells relative to 5FU alone. All statistical results were repeated for three times. * *P*<0.05; ** *P*<0.01; *** *P*<0.001

In a wound healing experiment, we found that MGC803 cells in the control group exhibited rapid migration within 24 hours after wounding, filling in half of the scratch area. The mobility of the 3.3 μmol/L 5FU groups at 24 hours after wounding was almost the same as that of the control group, while the wound healing of the 70 μmol/L loganetin group and the combination group was significantly reduced (Figure 3A). HGC27 cells in the control group migrated rapidly 24 hours after wounding, and half of the scratch area was filled. However, the wound healing of cells treated with 2.5 μmol/L 5FU or 70 μmol/L loganetin at 24 hours was partially reduced, while that of the combination group was more obvious (Figure 3B). Transwell assays were then carried out to analyse the invasive capabilities of HGC27 and MGC803 cells treated with these agents, revealing that the combination of 5FU and loganetin significantly decreased the invasiveness of HGC27 and MGC803 cells (Figure 3C).

### 3.4 The effects of the combination of 5FU and loganetin on EMT and stem‐like properties were attenuated by Wnt/β‐catenin signalling inhibition

**FIGURE 4:**
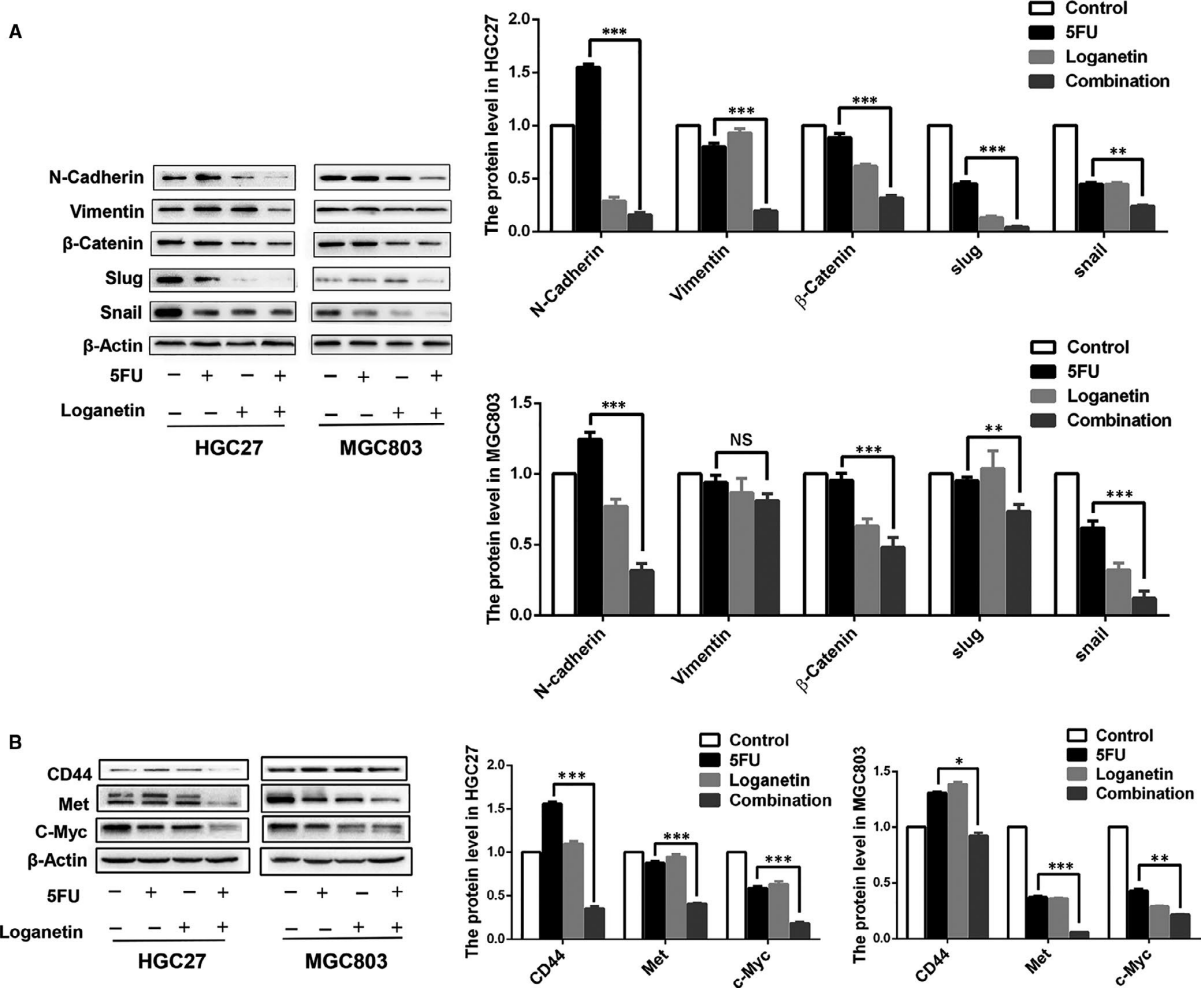
5FU and loganetin in combination inhibited the expression of EMT markers. (A) Western blotting assessment of the protein levels of EMT markers in HGC27 and MGC803 cells treated with 5FU (3.33 μM/ 2.5 μM), loganetin (70 μM) or both for 24h. (B) Western blotting assessment of the protein levels of Wnt target genes in HGC27 and MGC803 cells treated with 5FU (3.33 μM/ 2.5 μM), loganetin (70 μM) or both for 24h. All statistical results were repeated for three times. *P<0.05; ** P<0.01; *** P<0.001

To further examine whether EMT‐related molecular changes occurred upon treatment of cells with 5FU, loganetin or a combination (3.33 μmol/L 5FU and 70 μmol/L loganetin for HGC27 cells, 2.5 μmol/L 5FU and 70 μmol/L loganetin for MGC803 cells), we assessed the expression of EMT markers. After chronic exposure of cells to 5FU and loganetin, the protein levels of the EMT markers N‐cadherin, Vimentin, β‐catenin, slug and snail were decreased (Figure 4A). In many cancers, activating the Wnt/β‐catenin pathway could promote EMT and stem‐like properties.^22^ To further investigate the synergistic mechanism by which 5FU and loganetin inhibited stem‐like properties, migration and invasion of HGC27 and MGC803 cells, we next assessed whether 5FU and loganetin alone or in combination will affect this pathway in GC cells. After treatment of GC cells with 5FU and loganetin, several key proteins in the WNT/β‐catenin signalling pathway and its downstream targets including c‐myc, Met and CD44 were down‐regulated (Figure 4B). These experiments revealed that the combination of 5FU and loganetin could inhibit the proliferation, stem‐like properties, migration and invasion of GC cells, and WNT/β‐catenin signalling pathway may be partially involved in this process.

### 3.5 5FU and loganetin mediate synergistic inhibition of Wnt/β‐catenin signalling

**FIGURE 5:**
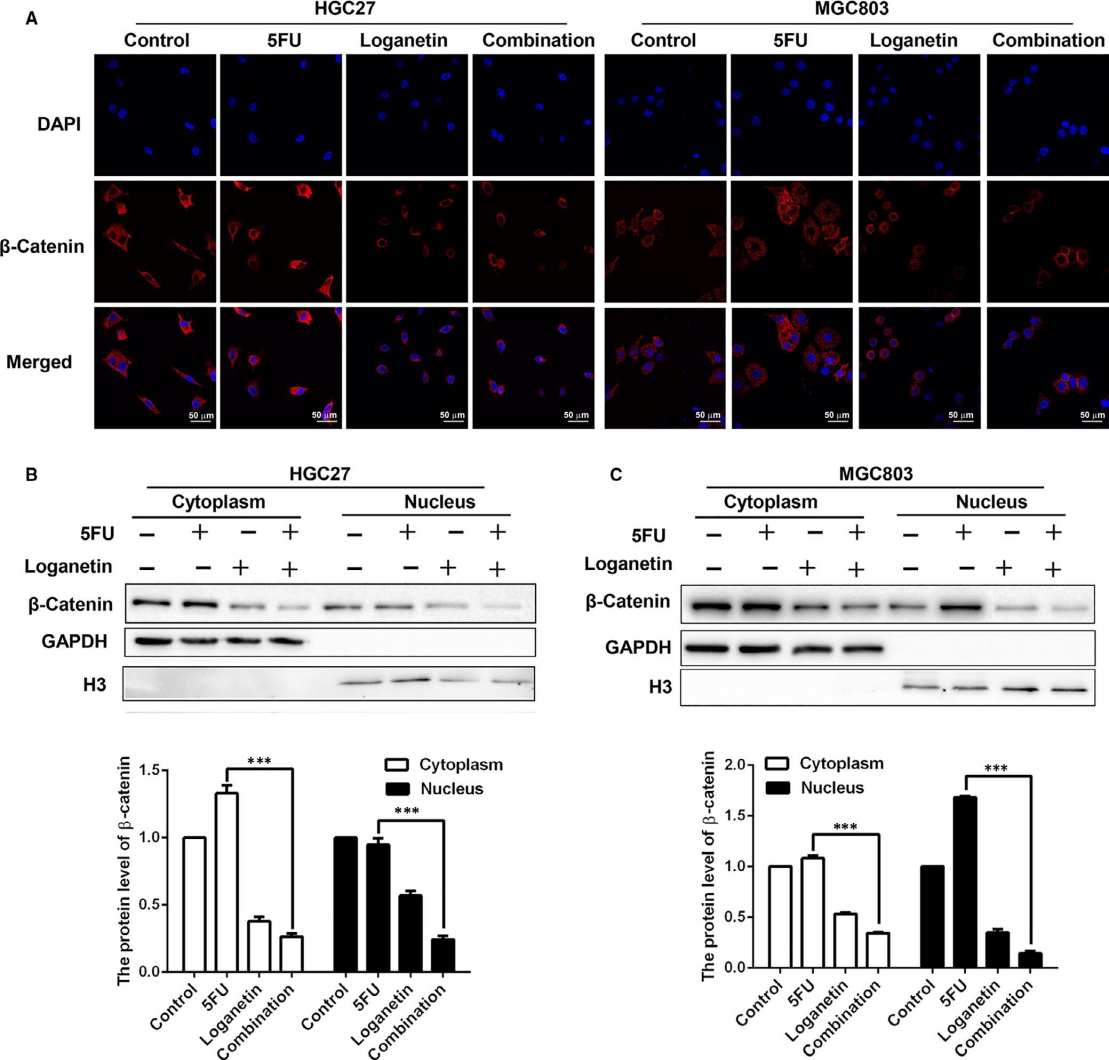
5FU and loganetin in combination affected the EMT and stem‐like properties via suppressing Wnt/β‐catenin signaling. (A) HGC27 and MGC803 cells were exposed to 5FU (3.33 μM/ 2.5 μM), loganetin (70 μM), or both for 24h. Immunofluorescence staining of β‐catenin expression in HGC27 and MGC803 cells was then conducted. Nuclei were counterstained with DAPI. Experiments were repeated three times. Immunoblots measuring cytoplasmic and nuclear β‐catenin in 5FU, loganetin, or combination treated HGC27 (B) and MGC803 cells (C). All statistical results were repeated for three times. *** *P*<0.001

To test whether WNT/β‐catenin signalling was significantly disrupted in HGC27 and MGC803 cells after treatment with 5FU, loganetin or a combination, we performed immunofluorescent staining for β‐catenin in both cell types. The combination of 5FU and loganetin treatment in HGC27 and MGC803 cells decreased the β‐catenin nuclear accumulation, as compared to controls or 5FU alone (Figure 5A). Western blotting further confirmed that GC cells treated with a combination of 5FU and loganetin exhibited reduced the nuclear and cytoplasmic β‐catenin level, as compared to control or 5FU‐treated cells (Figure 5B,C). Upon Wnt signalling activation, β‐catenin phosphorylation is decreased, and protein stability is thereby increased, leading to its cytoplasmic accumulation and nuclear transit, where it can interact with TCF and lymphatic enhancer binding factors to regulate the expression of target genes.^23^ Taken together, these results reveal that the combination of 5FU and loganetin can inhibit the WNT/β‐catenin signalling pathway and reduce the accumulation of β‐catenin in the cytoplasm and nucleus, thereby disrupting the process of GC.

### 3.6 Synergic anti‐cancer activity of 5FU and loganetin in vivo

**FIGURE 6:**
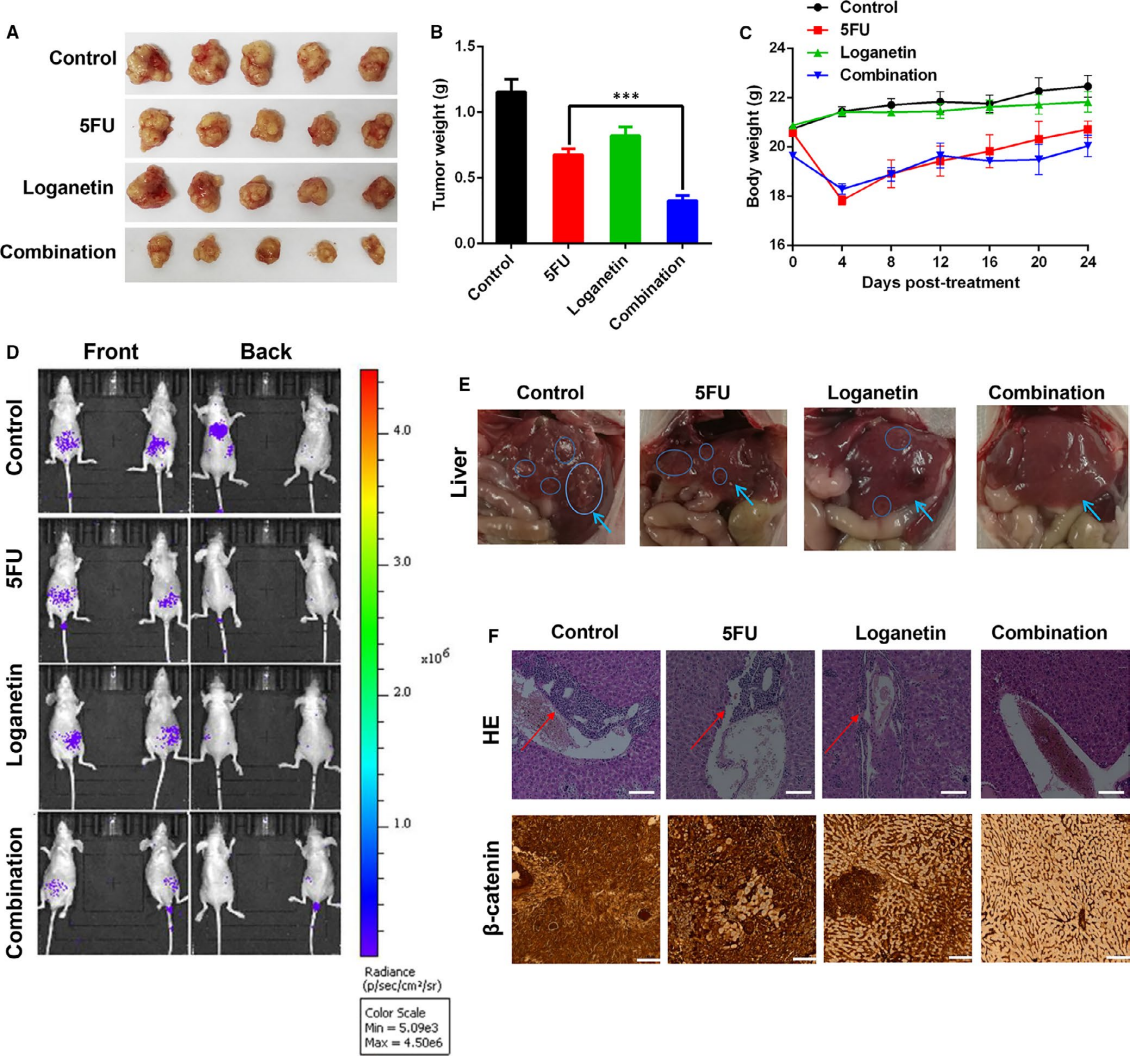
Anti‐tumor activity of the combination of 5FU and loganetin in vivo. (A) Representative images of tumors from each group on day 30. Tumor weight (B) and body weight (C) for MGC803 xenografts treated with 5FU, loganetin, or both. n=5. *** *P*<0.001. (D) After nude mice received a tail vein injection of HGC27‐luc cells (2 × 10 6 cells/ nude mice), the distribution of GC cells in these mice was observed via an in vivo fluorescent imaging system. (E and F) Representative histology of hepatic metastases of HGC27‐luc cells following tail vein injection in nude mice. HE: Scale bar,100um; IHC (β‐Catenin): Scale bar, 500um

To assess how 5FU, loganetin and combinational treatment affected GC xenografts in vivo, MGC803 cells were subcutaneously injected into nude mice. We found that 5FU was able to attenuate xenograft growth. However, loganetin on its own achieved a poor attenuation of xenograft growth. Interestingly, the combination of 5FU and loganetin could effectively inhibit xenograft growth compared with either agent alone (Figure 6A,B). Relative to the control, the body weight of nude mice treated with 5FU was significantly decreased (Figure 6C). The weight changes of nude mice in the loganetin treatment group and the control group were consistent (Figure 6C). To study the effects of 5FU, loganetin and the combination on GC metastasis, nude mice were administered a tail vein injection of HGC27‐luc cells. This experiment revealed that compared with 5FU and loganetin alone, the combined treatment inhibited the liver metastasis of GC more effectively (Figure 6D‐F).

## 4 DISCUSSION

Chemotherapy is vital to cancer treatment, and advanced GC is commonly relied on adjuvant or neoadjuvant therapy.^24^ However, chemotherapy has its own limitation, since it could promote tumour cell metastasis via regulating tumour microenvironment while killing the tumour cells.^25^, ^26^, ^27^ Furthermore, relapse frequently occurs after chemotherapy in advanced GC patients that is due to the development of drug resistance. Therefore, overcoming such resistance might achieve durable anti‐tumour responses.^28^

Chemotherapeutic reagents combination is often used to overcome the shortcomings of 5FU alone. One study found that the presence of folic acid cofactors could effectively increase the stability of 5FU, improving its anti‐tumour efficacy.^29^ Another study revealed that 5FU‐resistant xenograft tumours were reduced in size significantly when 5FU was combined with an inhibitor of glycogen synthase kinase 3 beta (GSK‐3β).^30^ Here, we found that 5FU alone had considerable limitations on suppressing the EMT and stem‐like properties of GC cells. Therefore, the combination of 5FU and other drugs might be an effective strategy to improve the anti‐tumour effect of 5FU. In the current study, we observed that loganetin was able to significantly inhibit GC cell growth and metastasis. Furthermore, the combination of 5FU and loganetin exhibited significant synergy, including inhibition of the growth, stem‐like properties, migration and invasion of GC cells.

*Cornus officinalis* is a commonly used traditional Chinese medicine,^12^ with loganin as its main active ingredient.^15^ While the biological activity of loganetin is poorly understood, loganin has been widely used.^31^ Recent work has revealed that loganetin protects against kidney injury in a TLR4‐dependent manner.^20^ However, the role of loganetin that plays in cancer was not previously known. Our data revealed that loganetin exhibits clear anti‐tumour efficacy (Figure 1B).

Stem‐like cell is an important cause of tumour progression.^32^ CD44 is the marker for GC stem‐like cells,^21^ and CD44^+^ GC cells reportedly exhibit more profound oncogenic potential in vitro and in vivo.^33^ In our study, stem‐like cells were enriched in the 5FU treatment group, while the combination of 5FU and loganetin had a synergistic inhibitory effect on this phenotype. Therefore, we proposed that the limitation of 5FU against stem‐like GC cells might contribute to its drug resistance, while loganetin can reverse this resistance. Compared with the 5FU treatment alone, the combination treatment significantly down‐regulated stemness‐related genes and proteins in stem‐like GC cells, which suggests that the combination of 5FU and loganetin exhibits synergistic anti‐tumour effects. Both the EMT and stem‐like properties are extremely important for tumour metastasis.^34^ We found that 5FU and loganetin together inhibited GC cell migration and invasion and significantly down‐regulated the expression of the EMT markers including N‐cadherin, Vimentin, β‐catenin, slug, as well as snail in HGC27 and MGC803 cells. These in vitro data suggested the combination of 5FU and loganetin could inhibit the EMT of GC cells, which was consistent with our in vivo results. Although the chemotherapeutic combination of 5FU for GC treatment has been previously studied,^35^ this is the first time to manifest the synergetic effect of loganetin with 5FU to inhibit the stem‐like properties and EMT of GC cells.

Wnt/β‐catenin signalling is an active driver of EMT and stemness in a variety range of cancers.^22^ Further, Wnt/β‐catenin signalling and nuclear β‐catenin accumulation are important causes of tumour development.^36^ We explored whether 5FU and loganetin, alone or in combination, could affect the Wnt/β‐catenin pathway in GC cells. Several Wnt/β‐catenin signalling pathway‐associated key proteins and its downstream targets, including c‐myc, Met, and CD44, were down‐regulated after 5FU and loganetin treatment in HGC27 and MGC803 cells. Moreover, the combination of 5FU and loganetin in HGC27 and MGC803 cells reduced nuclear β‐catenin accumulation, as compared to the control or 5FU alone. Therefore, the Wnt/β‐catenin pathway may play an important role in the synergistic mechanism of 5FU in combination with loganetin for the treatment of GC. However, the specific underlying mechanisms remain to be elucidated and will be the focus of our future investigations.

In summary, loganetin could not only efficiently inhibit GC cells alone, but also synergistically enhanced the anti‐tumour effect of 5FU on inhibiting GC cell stemness, migration and invasion. As such, our research provided preclinical evidence supporting the use of loganetin in cancer therapy, especially in combination with 5FU for the synergistic treatment of GC.

## CONFLICT OF INTEREST

There were no competing interests among all authors and their affiliations.

## AUTHOR CONTRIBUTION

**Huaixiang Zhou:** Investigation (equal); Writing‐original draft (equal). **Xiaoge Hu:** Writing‐review & editing (equal). **Na Li:** Investigation (equal). **Guangyan Li:** Investigation (equal). **Xiaotian Sun:** Writing‐review & editing (equal). **Feimin Ge:** Investigation (equal). **Jiahong Jiang:** Writing‐review & editing (equal). **Jingchun Yao:** Conceptualization (equal). **Dongsheng Huang:** Conceptualization (equal). **Liu Yang:** Conceptualization (equal); Writing‐original draft (equal); Writing‐review & editing (equal).

## Supporting information

Table S1‐S2

## Data Availability

The data that support the findings of this study are available in the supplementary material of this article.
