# Supplementary material for: Loganetin and 5‐fluorouracil synergistically inhibit the carcinogenesis of gastric cancer cells via down‐regulation of the Wnt/β‐catenin pathway
Source: J Cell Mol Med. 2020 Oct 24;24(23):13715–26. doi: 10.1111/jcmm.15932 (PMC7754039; doi:10.1111/jcmm.15932)
Supplement: Supplementary file 1 — Table S1‐S2 [file JCMM-24-13715-s001.docx]

**Supplementary material**

**Table S1 Primers were used for real-time quantitative PCR experiment**

| Gene | qPCR forward primer (5’-3’) | qPCR reversed primer (5’-3’) |
| --- | --- | --- |
| *OCT4* | TGAAGCTGGAGAAGGAGAAGCTG | TCTTTCTGCAGAGCTTTGATGTCCT |
| *Nanog* | TTCCTTCCTCCATGGATCTG | TCTGCTGGAGGCTGAGGTAT |
| *SOX* | ACACCAATCCCATCCACACT | GCAAACTTCCTGCAAAGCTC |
| *Bmil* | AGAGATCGGGGCGAGACAAT | TTGCTGGTCTCCAGGTAACG |
| *β-Catenin* | GAGTGCTGAAGGTGCTATCTGTCTG | TTCTGAACAAGACGTTGACTTGGA |
| *GAPDH* | GATTTGGTCGTATTGGGCG | TGGAAGATGGTGATGGGAT |

**Table S2 CI values of 5FU and loganetin**. CI values were calculated at different ratio concentrations of 5FU and loganetin in HGC27 and MGC803 cells by CalcuSyn.

| MGC803 | | |  | HGC27 | | |
| --- | --- | --- | --- | --- | --- | --- |
| 5FU (µM) | Loganetin (µM) | CI |  | 5FU (µM) | Loganetin (µM) | CI |
| 1.11  3.33  10  30  90 | 40  70  100  130  160 | 0.43  0.54  0.76  0.48  0.37 |  | 1.25  2.5  5  10  20 | 40  70  100  130  160 | 0.60  0.62  0.48  0.51  0.84 |

Notes: Synergism: (CI=0.3-0.7), Moderate synergism: (CI=0.7-0.85).
